# Routine Opt-Out HIV Testing Strategies in a Female Jail Setting: A Prospective Controlled Trial

**DOI:** 10.1371/journal.pone.0007648

**Published:** 2009-11-25

**Authors:** Ravi Kavasery, Duncan Smith-Rohrberg Maru, Joshua Cornman-Homonoff, Laurie N. Sylla, David Smith, Frederick L. Altice

**Affiliations:** Yale University School of Medicine, Section of Infectious Diseases, AIDS Program, New Haven, Connecticut, United States of America; University of Sao Paulo, Brazil

## Abstract

**Background:**

Ten million Americans enter jails annually. The objective was to evaluate new CDC guidelines for routine opt-out HIV testing and examine the optimal time to implement routine opt-out HIV testing among newly incarcerated jail detainees.

**Methods:**

This prospective, controlled trial of routine opt-out HIV testing was conducted among 323 newly incarcerated female inmates in Connecticut's only women's jail. 323 sequential entrants to the women's jail over a five week period in August and September 2007 were assigned to be offered routine opt-out HIV testing at one of three points after incarceration: immediate (same day, n = 108), early (next day, n = 108), or delayed (7 days, n = 107). The primary outcome was the proportion of women in each group consenting to testing.

**Results:**

Routine opt-out HIV testing was significantly highest (73%) among the early testing group compared to 55% for immediate and 50% for 7 days post-entry groups. Other factors significantly (p = 0.01) associated with being HIV tested were younger age and low likelihood of early release from jail based on bond value or type of charge for which women were arrested.

**Conclusions:**

In this correctional facility, routine opt-out HIV testing in a jail setting was feasible, with highest rates of testing if performed the day after incarceration. Lower testing rates were seen with immediate testing, where there is a high prevalence of inability or unwillingness to test, and with delayed testing, where attrition from jail increases with each passing day.

**Trial Registration:**

ClinicalTrials.gov NCT00624247

## Introduction

Over 2.3 million people, or one in every 100 American adults, are incarcerated and their initial interface with the correctional system is usually via jail.[1], [2] The prevalence of HIV infection in the United States is several-fold greater in correctional settings than in the general population. [3] Jails and prisons thus serve as important sites for HIV testing and treatment. [4], [5], [6], [7] The Centers for Disease Control and Prevention's (CDC) recent recommendation to implement routine opt-out HIV testing in all healthcare settings, including jails, has not been achieved due to logistical, financial and legal constraints.[8] Systematic solutions to logistical constraints within correctional settings, however, provides an important opportunity to advance our public health goals and expand access to HIV services for this vulnerable population.[9], [10], [11]

Jails interact with a large number of individuals at risk for HIV infection and pose unique logistical and health-related constraints that impact HIV testing strategies. [12] Jails, compared to prisons, are characterized by higher rates of turnover,[13] shorter stays, a higher prevalence of acute intoxication and withdrawal, and a higher number of inmates presenting with uncontrolled mental illness, recent HIV risk behaviors [14], [15] and suicidal behavior. [16], [17], [18], [19] Suicide incidence is three-times greater in jails than in prisons, with nearly a quarter taking place within 48 hours of admission.[20], [21]

Given the high attrition rate in jails, a major logistical challenge to implementing routine opt-out HIV testing is selecting the optimal time to conduct testing.[22] Newly incarcerated inmates might be too intoxicated or psychologically distressed to reliably consent to or opt out of routine testing, and may be unprepared to consider and respond to the consequences of a preliminary positive HIV test result.[12], [23] Likewise, the public health challenge with postponing HIV testing is that many individuals experience relatively short stays in jail and will be released before being tested.[24], [25]

Therefore, the objective of this study was to evaluate the optimal time to conduct routine opt-out HIV testing of newly incarcerated jail inmates in a manner that maximized the number of individuals capable of consenting and willing to be tested.

## Methods

The protocol for this trial and supporting CONSORT checklist are available as supporting information; see Checklist S1 and Protocol S1.

### Ethics Statement

This study was approved by the Institutional Review Board at Yale University and by the Connecticut Department of Correction Research Committee.

### Design Overview

Over a 5-week period starting August 22, 2007, all 323 consecutive, newly incarcerated female inmates were offered routine opt-out HIV testing after being sequentially assigned to one of three study arms upon admission to the facility: 1) ‘immediate’ (during a mandatory initial medical screen the night of admission); 2) ‘early’ (during a required physical exam the following evening); or 3) ‘delayed’ (7 days after arrival to the facility). Decisions about timing for routine opt-out HIV testing were based upon previous surveys of correctional and medical professionals as well as from experts in the field of HIV testing in correctional settings. These three time points were chosen to coincide with other routine healthcare activities at the jail in order to simulate the future implementation of a routine opt-out HIV testing protocol.

### Setting and Participants

This prospective, controlled trial was conducted at York Correctional Institution in Niantic, Connecticut, the state's sole correctional facility for women. Intake involves both sentenced and pre-trial detainees. The average daily census is 1641 inmates. Similar to other jails, a brief, standardized medical and psychiatric assessment is routinely conducted on all inmates, including medical, sexual, and drug-use histories immediately upon arrival. Testing for pregnancy, opioids, tuberculosis and acute medical conditions is routinely conducted. Inmates maintained on or experiencing opioid withdrawal symptoms are provided a methadone taper. The evening following admission, a routine physical examination, including Papanicolaou smear and phlebotomy, occurs in all new inmates remaining within the facility. [26] Voluntary HIV testing is available by medical referral or by self-request and often involves a waiting list. Inmates with self-reported HIV risks within the previous 90 days are deferred testing. Newly confirmed HIV positive test results are reported to the Connecticut Department of Public Health.

As part of this study, all newly incarcerated inmates were sequentially approached for competency and HIV testing and sequentially assigned to one of the three study groups. Eligibility to be HIV tested required demonstration of competency by: 1) clinician-confirmed ability to demonstrate knowledge of the risks, benefits, and consequences of HIV testing in accordance with the MacArthur Competence Assessment Tool for Clinical Research [MacCAT-CR][27]; and 2) no self-reported suicidal ideation or evidence of mental instability.

### Intervention

For each testing group, the inmate was approached with the following scripted statement: “As part of your regular medical care, HIV testing can now be done using an oral swab that you swipe across your gums. You can receive your results after 20 minutes. Would you like to be tested at this time?” If the inmate responded affirmatively, she was instructed to self-administer the oral HIV test by the clinical staff in the ‘immediate’ and ‘early’ test groups as part of routine clinical activities in order to simulate how routine opt-out HIV testing would be performed if not embedded within a complicated research study. On day 7, research personnel oversaw the verbal consent and self-administration procedures using the same process. All subjects were instructed that HIV results require minimal waiting. Anyone not wanting to know HIV test results was not swabbed. If the inmate agreed to be swabbed and tested, she *subsequently* met with a research assistant who discussed two written informed consents – one for study participation and one for HIV testing (legislatively mandated). Inmates who initially agreed to be swabbed but refused to provide both written consents did not have their HIV swabs tested and these specimens were immediately discarded. These individuals, along with anyone not wanting testing were informed voluntary HIV testing was available through self-referral from an HIV counselor. Those who self-identified as being HIV-infected were not swabbed.

### Outcomes and Follow-up

Oral swab testing was conducted onsite using the OraQuick ADVANCE® rapid HIV-1 antibody test [sensitivity: 99.3% (98.4–99.7), specificity: 99.8% (99.6–99.9)].[28], [29] The primary outcome was the proportion of individuals in each assigned group that provided verbal consent to be swabbed for HIV testing. Individuals were not swabbed for HIV testing if they were physically not available (e.g., released from jail, at court, attorney visits, too ill), were deemed medically incompetent to provide consent, or opted out of HIV testing. The primary outcome, using a public health perspective, was analyzed using an intention-to-treat (ITT) approach and included all 323 inmates admitted to the jail during the study period, as assigned. In our intention-to-treat analysis, we assessed whether an inmate was swabbed, regardless of whether they subsequently agreed to take part in the research protocol. Any subject for whom swab results were missing were deemed “failure to swab” in the analysis; however, there were no missing data in the final database. A secondary outcome, to assess individual acceptability of HIV testing, was the proportion of inmates who agreed to HIV testing among those still under correctional supervision at the time that testing was offered.

Pre-test counseling was not provided. Subjects who received a preliminary positive test result were immediately referred for phlebotomy for confirmatory testing with Western blot. Certified HIV counselors provided preliminary-positive post-test counseling and confirmatory results; study staff delivered negative results.

As an additional secondary analysis, inmates deemed competent to receive testing who provided written consent were asked about previous HIV testing experiences, attitudes toward HIV testing in jail settings and were also administered a series of standardized instruments: Clinical Opiate Withdrawal Scale [COWS], [30] Clinical Institute Withdrawal Assessment of Alcohol Scale [CIWA-Ar], [31] and the Kessler 6-Item Psychological Distress Scale (K6).[32]

To determine if the three testing groups differed with regard to social and demographic characteristics, the Connecticut Department of Correction (CTDOC) database was queried to abstract demographic characteristics [age and race (defined by CTDOC)], type of charge and bond value. No unique identifiers were provided. Low likelihood of early release was defined as a bond value ≥$5,000, sentencing >30 days, immigration or federal charges, or no bond allowed. High HIV-risk charges were considered to be any charges directly related to prostitution or drugs.

### Statistical Analysis

The primary outcome was the proportion of women in each testing group who were orally swabbed and provided verbal consent to receive rapid HIV testing. Using two-sided Chi-Square tests for assessing three pair-wise differences between the different study arms and applying Bonferroni's correction (i.e., alpha = 0.0166 for each comparison), we sought to collect 97 patients in each arm to achieve 80% power to detect a 22% difference between arms given a baseline uptake of 60%. Comparisons of demographic, correctional and refusal characteristics were conducted using two-sided Chi-Square tests (alpha = 0.05).

After calculating the bivariate associations with the primary outcome, a multiple logistic regression model was developed to predict the likelihood of being swabbed using the available subject characteristic variables. The Akaike information criterion (AIC) was used to assess model fit; lower AIC values indicate a better balance of parsimony and explanation of variance. In conjunction with AIC, a p-value of 0.30 was used to enter and leave the model. The optimal model was chosen as the convergence of the forward and backward models, with consideration of parsimony and plausibility. The two-sided Wald's test (alpha = 0.05) was used to assess significance of each of the variables. All statistical analyses were conducted using SAS, version 9.1.3 (SAS Institute).

## Results

The baseline characteristics of the study population are shown in Table 1. During the study period, 323 newly incarcerated women were sequentially assigned to the following testing groups: ‘immediate’ (N = 108, the night of admission), ‘early’ (N = 108, the following evening), and ‘delayed’ (N = 107, 7 days later). The three study groups did not differ significantly with respect to any of the social and demographic characteristics assessed.

**Table 1:** Baseline Characteristics of the Study Population (n = 323).

Characteristics	Subcategory	Value (%)
Age (mean years; SD)		33.6 (9.8)
Length of Current Incarceration (median days; IQR)		28 (7–94)
Race	Hispanic	53 (16)
	Black	104 (32)
	White/Other	166 (51)
Education	High School Graduate	201 (62%)
	Not a High School Graduate	122 (38%)
Likelihood of Early Release*	High	115 (36)
	Low	208 (64)
Type of Charge	Drug- or Prostitution-Related	81 (25)
	Not Drug- or Prostitution-Related	242 (75)
Previous Incarcerations	Never Incarcerated	117 (36)
	Incarcerated Previously	206 (64)
	Mean Number of Previous Incarcerations (N; SD)	1.9 (2.4)
Medical Insurance	Yes	120 (37)
	No	203 (63)
Urine Toxicology	Negative for Opiates	242 (75)
	Positive for Opiates	81 (25)

* High: any charges directly related to prostitution or drugs.

Low: bond value ≥ $5000, bond sentencing >30 days, immigration or federal charges, or no bond.

**Figure 1:**
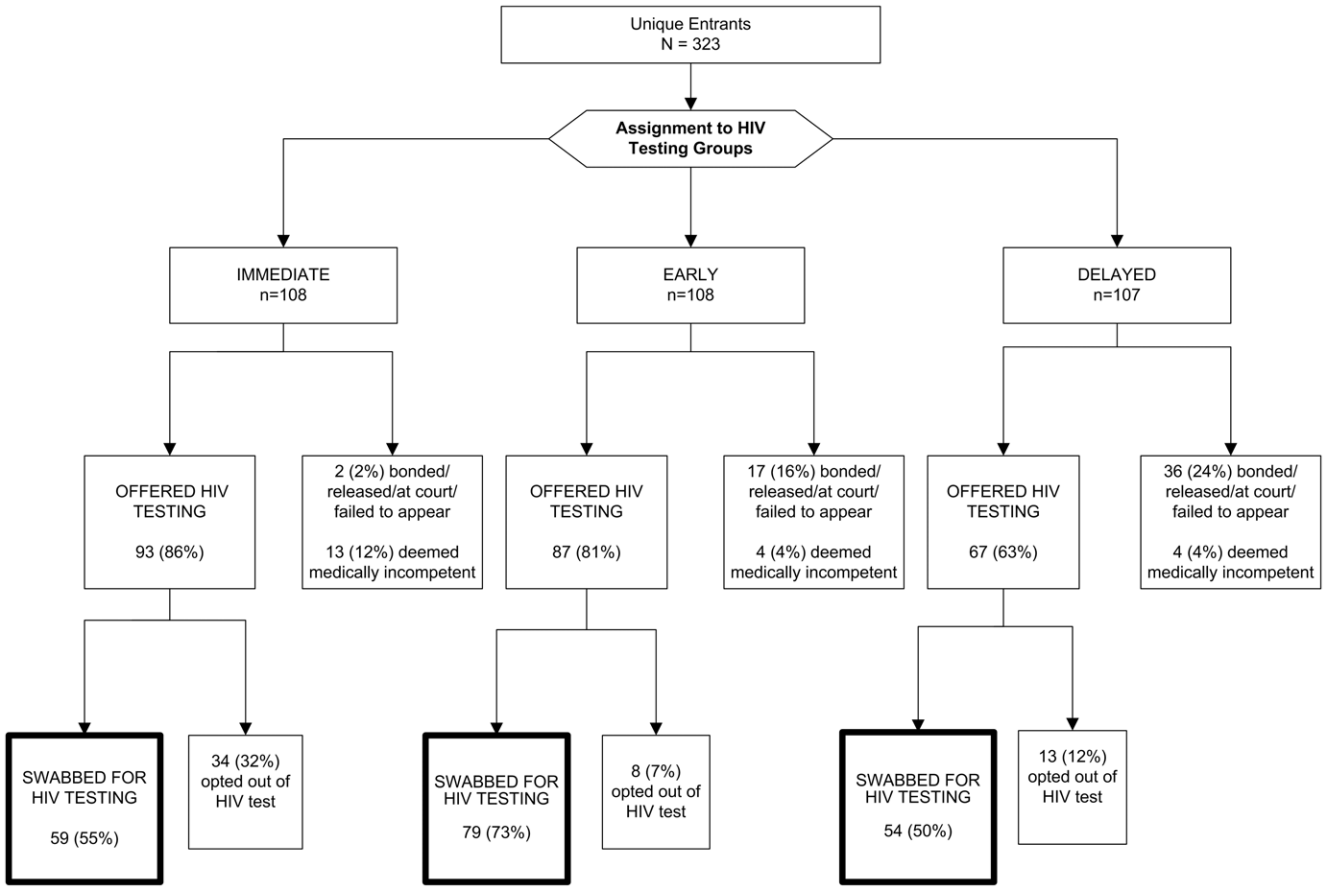
Disposition of Inmates Approached for HIV Testing.

**Figure 2:**
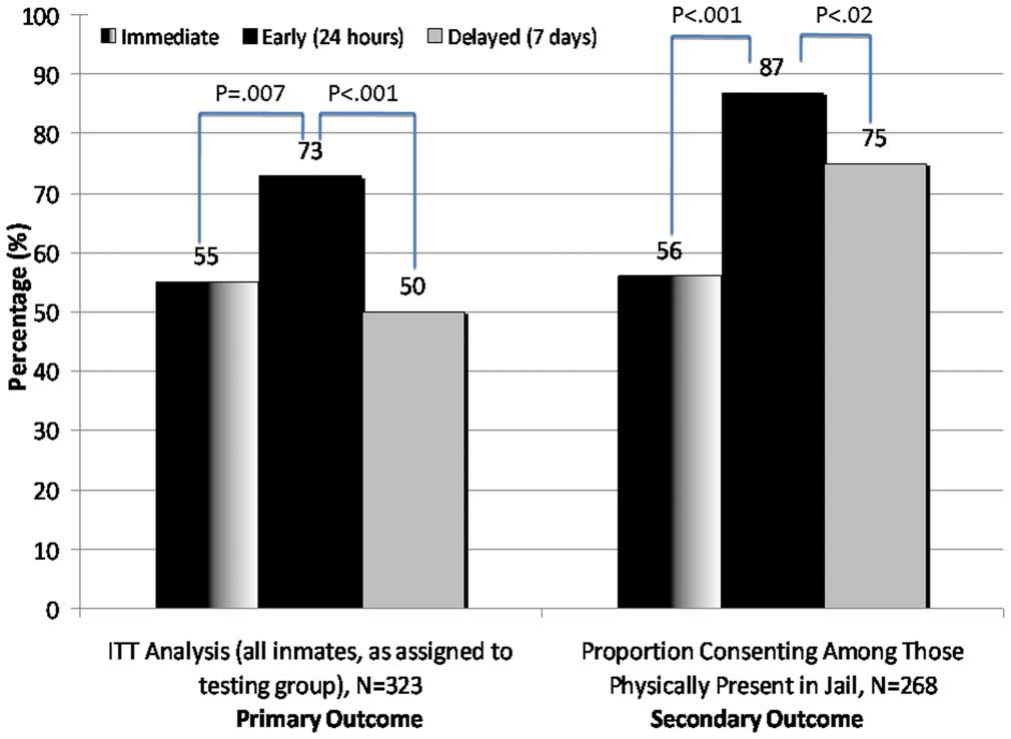
Rapid HIV Testing Swab Results by Assigned Testing Group.

The disposition of individuals approached for routine opt-out HIV testing in this trial is illustrated in Figure 1. Overall, 192 (59%) of 323 inmates assigned to testing groups provided verbal consent to be swabbed for HIV testing. For the primary outcome, 79 (73%) of those offered ‘early’ testing, received an HIV test, compared to 59 (55%) assigned to the ‘immediate’ and 54 (50%) assigned to the ‘delayed’ testing groups (Figure 2). The early testing group was significantly more likely to be tested than both the immediate group (OR = 2.3; 95% CI = 1.3–4.0; p = 0.007) and the delayed group (OR = 2.7; 95% CI = 1.5–4.7; p = 0.0007). The proportion swabbed in the immediate and delayed testing groups, however, did not differ (OR = 1.2; 95% CI = 0.7–2.0; p = 0.54). To assess the individual acceptability of HIV testing, 268 subjects were physically present within the jail at the three time points when routine opt-out testing was made available (see Figure 2). Acceptability was highest for the early testing group (N = 79/91, 87%), compared to 76% (N = 54/71) in the delayed and 56% (N = 59/106) in the immediate testing group (p<0.05 for all comparisons).

Stratified by testing group, the reasons that inmates were not swabbed are depicted in Table 2. In the ‘immediate’ group (N = 108), 12 (11%) were medically incompetent to consent, compared with only 4 (4%) in each of the ‘early’ (N = 108) and ‘delayed’ (N = 107) testing groups. This difference was significant (OR = 3.2; 95% CI = 1.3–8.2; p = 0.009; not depicted in Table 2). In the ‘delayed’ testing group, 36 (34%) did not appear for testing compared with 4 (4%) in the ‘immediate’ and 17 (16%) in the ‘early’ testing groups (OR comparing delayed to other: 4.7; 95% CI: 2.6–8.6; p = 0.000001; not depicted in Table 2). The most common reasons for failing to be available for testing included being released from the facility (either paid bond or released from court), appearing in court that day, or rarely, logistical barriers within the jail setting that prevented movement within the facility. Among the 54 competent subjects who declined testing, 27 (54%) stated they did not perceive themselves at risk, 10 (19%) declared they were already HIV-infected (all were confirmed by medical record review), and 8 (15%) stated they were too tired, fearful of testing, or experiencing withdrawal.

**Table 2:** Reasons Inmates were not Swabbed for HIV Testing.

Reason	Immediate Group	Early Group	Delayed Group
	(49 not swabbed of 108)	(29 not swabbed of 108)	(53 not swabbed of 107)
Bonded/Released/At Court, n (%)	4 (8)	17 (59)	36 (68)
Refused/Declined Swab or Study Participation, n (%)	33 (67)	8 (27)	13 (24)
Medically Incompetent/Failed MacArthur, n (%)	12 (25)	4 (14)	4 (8)

**Figure 3:**
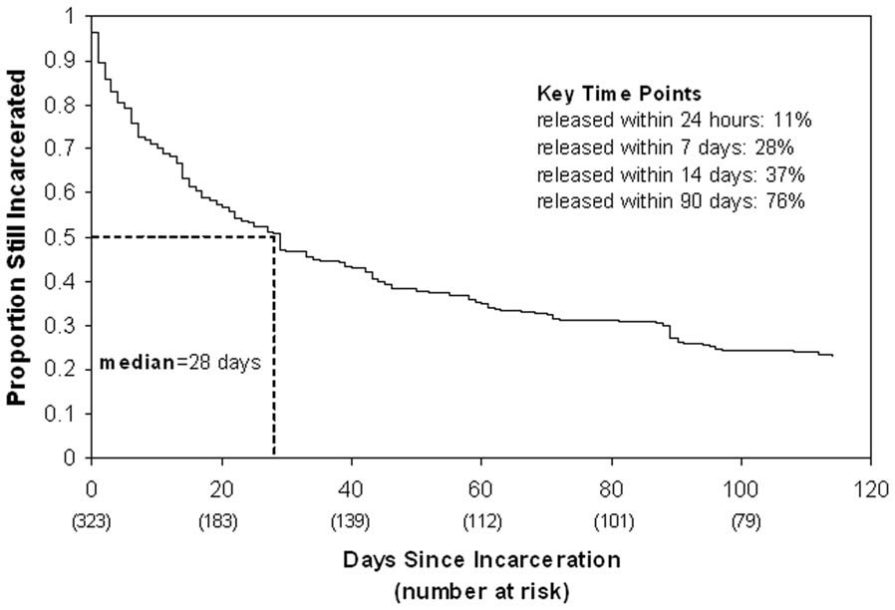
Time to Release Following Incarceration.

Figure 3 demonstrates the first attrition-decay curve from a jail expressed over time. The median duration of incarceration was 28 days; among the 323 subjects approached, 90 (28%) were no longer incarcerated after 7 days, 118 (37%) after 14 days, and 247 (76%) at 90 days after admission. The highest attrition rate was within the first 24 hours with 11% (n = 34) leaving the facility during this time. These individuals, compared to those who were released at later times, trended toward having less opiate-positive urine test results (11% vs. 26%, p = 0.06) and were less likely to be jailed for sex- or drug-related charges (11% vs. 26%, p = 0.06). They were also significantly less likely to have been previously incarcerated (43% vs. 66%, p = 0.009). Bivariate and multivariate analyses were conducted to determine predictors associated with being swabbed for HIV testing (Table 3). In the bivariate analysis, assignment to the ‘early’ testing group, younger age, low-likelihood of release, high HIV-risk charges, and being Hispanic were associated with being swabbed for HIV testing. In the multivariate analysis, assignment to the ‘early’ testing group (p<0.001), younger age (p = 0.01), and low likelihood of release (p = 0.01) remained significantly associated with being swabbed for HIV testing.

**Table 3:** Bivariable and Multivariable Predictors of Receipt of Swab.

	*Uptake Rates, n (%)*	*Bivariable OR (95% CI)*	*Bivariable p-value*	*Multivariable OR (95% CI)*	*Multivariable p-value*
Assigned day 0*	59 (55)	1.2 (0.7 to 2)	0.54	1.2 (0.7 to 2.1)	0.51
Assigned day 1*	79 (73)	2.7 (1.5 to 4.7)	0.0007	**2.7 (1.5 to 5)**	**0.0009**
Assigned day 7*	54 (50)	--Referrent--	--	--Referrent--	--
Age (yrs) at Entry**	--	0.7 (0.6 to 0.9)	0.01	**0.7 (0.6 to 0.9)**	**0.01**
Low Likelihood of Release	133 (64)	1.7 (1.1 to 2.7)	0.03	**1.9 (1.1 to 3.1)**	**0.01**
High Likelihood of Release	59 (51)	--Referrent--	--	--Referrent--	--
High HIV-Risk Offense	56 (69)	1.7 (1 to 3)	0.04	1.7 (1 to 3)	0.07
Low HIV-Risk Offense	136 (56)	--Referrent--	--	--Referrent--	--
Black	55 (53)	0.7 (0.4 to 1.1)	0.10	0.7 (0.4 to 1.2)	0.17
Hispanic	40 (75)	2.4 (1.2 to 4.7)	0.01	2 (1 to 4.1)	0.07
White/Other	97 (58)	--Referrent--	--	--Referrent--	--
Previous Incarceration	125 (61)	1.2 (0.7 to 1.8)	0.55	-Out of Model-	--
No Previous Incarceration	67 (57)	--Referrent--	--	-Out of Model-	--
Urine Opiate(+)	51 (63)	1.2 (0.7 to 2)	0.46	-Out of Model-	--
Urine Opiate(-)	141(58)	--Referrent--	--	-Out of Model-	--
High School Graduate	118 (59)	0.92 (0.6 to 1.5)	0.73	-Out of Model-	--
Not High School Graduate	74 (61)	--Referrent--	--	-Out of Model-	--
Has Medical Insurance	66 (55)	0.7 (0.5 to 1.2)	0.21	-Out of Model-	--
No Medical Insurance	126 (62)	--Referrent--	--	-Out of Model-	--

*OR Comparing day 1 or day 7, respectively to day 0.

**The calculated OR represents the added likelihood conferred by every 10 years of age.

Of the 192 individuals who were swabbed, 151 (79%) provided written consent to complete the entire study. Two additional participants failed to pass the MacArthur Competence Assessment Tool, leaving 149 (79%) individuals eligible to be HIV tested. Of these, 147 (99%) were HIV-negative and two had a preliminary-positive test result; both results were false-positive after obtaining confirmatory Western Blot testing. Thus, none of the 149 people tested were diagnosed as being HIV-infected. Two negative test results (one from the ‘immediate’ and one from the ‘early’ testing groups) were not delivered due to the inmate having left the facility.

Among the 149 subjects HIV-tested subjects that underwent standardized screening, 11 (7%) exhibited moderate or severe opioid withdrawal symptoms: three (7%) from ‘immediate’, eight (15%) from ‘early’ group, and none from the ‘delayed’ testing group. Ten (7%) individuals were deemed to have increased risk for alcohol withdrawal symptoms: three (7%) from ‘immediate’, seven (13%) from ‘early’, and none from the ‘delayed’ testing group. In addition, 50 (34%) of the 149 tested subjects had evidence of serious mental illness using the K6 psychological distress scale score: 11 (24%) from ‘immediate’, 22 (42%) from ‘early’, and 17 (33%) from the ‘delayed’ testing group. Nearly all (89%) of these 149 subjects self-reported having been HIV tested previously, but only 32% reported testing within the previous year. The most recent HIV testing had occurred previously at a community organization (n = 32, 21%), hospital (n = 30, 20%), or correctional facility (n = 29, 19%).

## Discussion

To our knowledge, this is the first prospective, controlled trial of routine opt-out HIV testing among female jail inmates, a population that typically experiences high rates of psychological distress, rapid turnover, and both acute intoxication and withdrawal upon admission. Previously, voluntary testing had been shown to have limited uptake rates; one multicenter study tested only 6% of ∼550,000 jail detainees using voluntary testing methods. [33] Two observational studies had suggested that routine opt-out HIV testing was feasible.[23] Our results confirm the feasibility of opt-out, routine rapid HIV testing in a jail setting and suggest that waiting until the evening after entry increases the number of individuals who receive HIV testing. This is likely due to optimizing the balance between allowing time for psychological and medical stabilization of the individual and expeditiously providing testing prior to individuals leaving the facility. The magnitude of these effects are of significant public health importance, in that 73% of those approached the evening after admission were swabbed for HIV testing, compared to 55% and 50% of those approached immediately or seven days post-entry, respectively. This benefit was seen despite the fact that 11% of inmates at this facility were released within the first 24 hours.

Similarly, individual acceptability of HIV testing was also highest among those in the early testing group. Of the 268 women physically present in the facility at the time they were offered, 87% of those approached the evening after admission verbally consented to testing, compared to 56% and 75% of those in the immediate and delayed testing groups, respectively. Testing inmates on the day of incarceration may be less optimal because these individuals are distraught from being arrested and tired from remaining in court or in a holding cell all day. The substantial increase in willingness to test 24 hours after admission may reflect acceptance of being incarcerated, in addition to having had a night of sleep. Though unclear from these data, acceptability decreased after remaining in the jail after 7 days, perhaps explained by the impact of peer pressure and/or recognition of potential stigma from HIV testing. Despite acceptability being slightly lower among those individuals approached for testing one week post-entry, a higher proportion consented than found in voluntary HIV testing programs in other correctional settings.[33], [34]

Because this study was restricted to a single, female correctional facility, the findings may not be generalizable to all jail settings. Not all jails provide routine clinical assessments the day following admission, and others may not provide any routine healthcare services at all.[35] Furthermore, large, metropolitan correctional facilities experiencing many-fold higher daily admissions may face additional logistical challenges in implementing testing as part of intake procedures. Finally, gender differences may also result in markedly different uptake rates of HIV testing among male inmates compared to females.

In this study, the most common reasons for not being swabbed for HIV testing included early release from the facility (presumably due to posting bond), failing to demonstrate medical competency to consent to testing, and choosing not to be HIV tested. On multivariate analysis, additional factors significantly associated with receiving HIV testing were younger age (conferring a 7% decrease in the likelihood of testing for every ten years of increasing age) and having bond set above $5,000 (conferring nearly a 2-fold reduced likelihood of being released). Ability or willingness to test was particularly important for testing in the ‘immediate’ group. Although almost a third of individuals assigned to ‘immediate’ testing refused testing (Figure 2), only 7% of those approached one day later chose to opt-out. Those assigned to the ‘immediate’ testing group were also 3-times more likely than either of the other groups to be medically or psychologically unable to consent to testing. One potential explanation for the higher rate of testing in the ‘early’ testing group, particularly compared to the immediate group, is that women may have gotten some rest, been initiated on medication-assisted protocols to treat opioid or alcohol withdrawal, or had become resigned to being in jail.

It was clear that the high-risk women in this study had not been adequately reached with HIV testing services. While 89% of those who consented to study participation reported being previously tested for HIV, only 30% had received an HIV test within the last year, per CDC recommendations for high-risk individuals.

Our protocol achieved a reasonable balance of personal autonomy and effectiveness so critical to achieving good outcomes in correctional settings. [12], [36] Using a routine opt-out HIV testing program, those individuals who perceive themselves at high-risk and are therefore fearful to test can still choose to not be tested. Likewise, those who don't perceive themselves to be at risk may fail to take advantage of the opportunity to receive testing. Over one-third of the 27 inmates who opted-out of HIV testing would otherwise be deemed at significant risk for HIV, supported by 4 (15%) testing positive for opiates and another 5 (19%) arrested for prostitution or drug-related charges. These data suggest that if an HIV testing strategy is to be implemented, it is important that reasons for refusal are properly addressed to optimize uptake of HIV testing among those who might be at highest risk or may not recognize their risk at all.

A major strength of the present study design was that it enabled us to accurately assess realistic acceptance for HIV testing in an ethical manner. Socially marginalized individuals, such as prisoners, may be leery about participating in research in coercive places like jails. [2], [37] We overcame this obstacle by asking jail-based clinicians to ask individuals to provide verbal consent to be HIV tested before referring them to research personnel to obtain written consent for study participation. Thus, this trial simulated what routine opt-out HIV testing within a clinical encounter in jail might look like and avoids biasing participant response during the encounter. Indeed, approximately 22% of those subjects who agreed to be swabbed for rapid HIV testing as part of routine intake procedures later refused to provide consent for study participation. In most cases, this was because of subjects' suspicion of being involved in research or because of the time involved in completing several interview instruments at a time when they were tired or did not feel well. The primary outcome of being swabbed for an HIV test, therefore, served as a better marker in this trial for acceptance of HIV testing than completion of the informed consent aspect of the study and thereby receiving an HIV test result.

Although this trial successfully demonstrated the feasibility of routine opt-out HIV testing in a jail, challenges remain to be addressed before routine opt-out HIV testing is implemented more widely in other jail settings. Daunting challenges remain to implement routine opt-out HIV testing upon intake at some of the largest and busiest jails. Several hundred people may be processed daily, with intake procedures taking place 24 hours a day.

One of the unresolved issues for routine opt-out HIV testing in jails is ensuring delivery of confirmatory HIV test results for those who test preliminarily-positive. In this trial, only two (0.6%) of the 323 women approached for testing received a preliminary-positive test result that required a confirmatory blood draw, the results of which often require up to a week to receive. Although both individuals in this trial were still incarcerated in the facility and therefore able to receive their confirmatory results a week later, there will be cases of release prior to receipt of results. Indeed, over one-quarter of the inmates in this study were released within seven days of entry. While we await more rapid, confirmatory testing technology, Western Blot testing remains the accepted standard. Therefore, establishing linkages to public health systems in the community is required to ensure case-finding after release and ensure delivery of confirmatory results.

We conclude that routine opt-out HIV testing in jails is feasible, with the highest testing yield occurring one day after incarceration. This approach balances the medical and psychiatric instability seen among those immediately upon incarceration with the high attrition rate demonstrated by those tested 7 days later. Notwithstanding the merits of answering the logistical question of when to HIV test, many other questions remain, including how to avoid repeat testing, costs associated with increased HIV testing and barriers associated with written informed consent.

## Supporting Information

Checklist S1 CONSORT Checklist
(0.06 MB DOC)

Protocol S1 Trial Protocol
(0.43 MB DOC)
