# Supplementary material for: Routine Opt-Out HIV Testing Strategies in a Female Jail Setting: A Prospective Controlled Trial
Source: PLoS One. 2009 Nov 25;4(11):e7648. doi: 10.1371/journal.pone.0007648 (PMC2777332; doi:10.1371/journal.pone.0007648)
Supplement: Protocol S1 — Trial Protocol (0.43 MB DOC) [file pone.0007648.s002.doc]

**
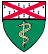
**

**YALE UNIVERSITY SCHOOL OF MEDICINE**

YALE NEW HAVEN HOSPITAL

**HUMAN INVESTIGATION COMMITTEE**

**Application to Involve Human Subjects in Research**

| **Title of Research Project:**  Early Versus Delayed Routine HIV Testing in Connecticut Jails | | | | | | |
| --- | --- | --- | --- | --- | --- | --- |
| **Principal Investigator:**  Frederick L. Altice, M.D. | | | | **Yale Academic Appointment:**  Director, HIV in Prisons Program;  Director, Clinical and Community Research;  Associate Professor of Medicine | | |
| **Campus Address:**  Yale University AIDS Program  135 College Street, Suite 323  New Haven, CT 06510-2283 | | | | | | |
| **Campus Phone:** 203.737.2883 | **Fax:** 203-737-4051 | | **Pager:** | | | **E-mail:** [raltice@aol.com](mailto:raltice@aol.com)  [frederick.altice@yale.edu](mailto:frederick.altice@yale.edu) |
| **Protocol Correspondent Name & Address:**  Ravi Kavasery, YSM I  c/o John Phifer  367 Cedar St, Rm 100  Yale School of Medicine  New Haven, CT 06510 | | | | | | |
| **Campus Phone:** 631-882-8804 | | **Fax:** | | | **E-mail:** [ravi.kavasery@yale.edu](mailto:ravi.kavasery@yale.edu) | |

## Section I: Principal Investigator/Faculty Advisor Agreement

As the Principal Investigator or Faculty Advisor of this research project, I certify the following:

- The information provided in this application is complete and accurate.
- That I assume full responsibility for the protection of human subjects and the proper conduct of the research.
- That subject safety will be of paramount concern, and every effort will be made to protect subjects’ rights and welfare.
- That the research will be performed according to ethical principles and in compliance will all federal, state and local laws, as well as institutional regulations and policies regarding the protection of human subjects.
- That all members of the research team will be kept apprised of research goals.
- That I will obtain approval for this research study and any subsequent revisions prior to initiation.
- That I will report to the HIC any serious injuries or other unanticipated problems involving risk to participants.

________________________________________

Signature Date

## Section II: Funding, Training and Protocol-Related Conflict of Interest

1. **Funding Source:** Please describe the funding source(s) for this study. Check all boxes that apply:

External* Department Name: Investigator Initiated:

Other: ______

*For externally funded studies, please supply the following information:

- - PI of Contract or Grant: Frederick L. Altice, M.D.
  - Funding Source: NIDA
  - Contract or Grant Title: K-24 Midcareer Investigator Award in Patient-Oriented Research
  - Contract or Grant #: K24-DA-017072

If using more than one funding source for this study, list all funding sources on an attached sheet. For grants: attach those sections of your grant application/agreement that pertain to the technical and human subject’s portion of this protocol.

1. **Human Subject Protection Training:** All investigators and study personnel (persons involved in the design and/or conduct of research involving human subjects) are required to complete human subject protection training (HSPT). This training requirement can be met through the Yale web-based program at <http://info.med.yale.edu/irbtraining/> or the NIH program at <http://www.cancer.gov/clinicaltrials/learning/page3>. Please note that investigators who have not completed this training requirement cannot participate in study activities until this training is completed.
2. **Conflict of Interest Statement:** All investigators and study personnel (those persons involved in the design and/or conduct of the research involving human subjects) are required to read a copy of the Yale Human Investigation Committee Policy on Protocol-Related Conflict of Interest (“HIC COI Policy” – see <http://info.med.yale.edu/hic/policy/index.html> ).  ***Please note that the HIC COI Policy addresses protocol-related conflict of interest, and is distinct from the annual disclosure required by the Yale University Policy on Conflict of Interest and Conflict of Commitment*.**

All investigators and study personnel are required to sign their name in the space provided below. Those who have answered “no” to all screening questions asked in the HIC COI Policy should indicate below that no Protocol-Related COI exists. Those who answered “yes” to any question in the HIC COI Policy should download a copy of the Protocol-Related Conflict of Interest Disclosure Form, which must be submitted to the HIC along with this Application.

|  | Name | **Signature ***** | Protocol-Related  COI? | HSPT Completed? | Affiliation |
| --- | --- | --- | --- | --- | --- |
| **Principal Investigator** | Frederick L. Altice, MD | 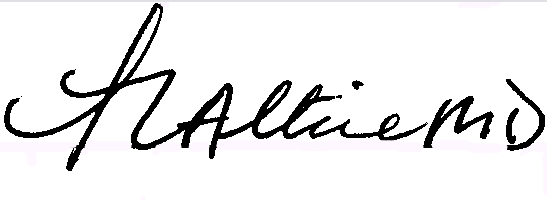 | Yes  No | Yes  No | Associate Professor, Yale School of Medicine |
| **Co-Investigator(s)** | n/a |  | Yes  No | Yes  No |  |
| **Study Personnel** | Douglas Bruce, MD, MA | 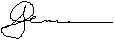 | Yes  No | Yes  No | Yale AIDS Program, Yale University School of Medicine |
|  | Ravi Kavasery |  | Yes  No | Yes  No | medical student, Yale School of Medicine |
|  | Duncan Smith-Rohrberg | 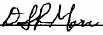 | Yes  No | Yes  No | MD/PhD student, Yale University |
|  | Ryan Schwarz |  | Yes  No | Yes  No | medical student, Yale School of Medicine |
|  | Laurie Sylla |  | Yes  No | Yes  No | International, Community Research Director, Yale AIDS Program |
|  | David Smith |  | Yes  No | Yes  No | Associate Community Research Director, Yale AIDS Program |
|  | James Taylor |  | Yes  No | Yes  No | Research Assistant |
|  | Nancy Manifold |  | Yes  No | Yes  No | Research Nurse |
|  | Jo Anne Mezger |  | Yes  No | Yes  No | Yale AIDS Program |
|  | Shu Chen |  | Yes  No | Yes  No | Yale AIDS Program |
|  | Joshua Cornman-Homonoff |  | Yes  No | Yes  No | Research Assistant |

Indicate under Affiliation whether the Investigator or Study personnel are part of the Yale Faculty or staff or part of the faculty or staff of a collaborating institution.

***** My signature here indicates that I have read and am in compliance and will continue to be with the HIC’s Protocol-Specific Conflict of Interest Policy.**

1. **Department Chair’s Conflict of Interest Statement (to be completed by the Chair of *each department* with which the Principal Investigator and co-investigators are affiliated and/or which the research affects):** Do you know of any real or apparent institutional conflict of interest (e.g., Yale University ownership of a sponsoring company) that might compromise this research?

Yes No

__________________________________________ ___________________________________

*Signature of Chair******** *Department*

*******My signature here indicates that I have read and am in compliance with the HIC’s Protocol-Related Conflict of Interest Policy. I further agree to submit a Protocol-Related Conflict of Interest Disclosure Form if I am aware of any real or apparent institutional conflict of interest.

**APPROVED FOR SUBMISSION TO HIC:**

__________________________________________ ________________________________ _______

*Signature of Primary Reviewer* *Please Print Name of Primary Reviewer Date*

**For HIC Use Only**

**__________________________ ____________________________________**

**Date Approved Human Investigation Committee**

Protocol is valid until: ___________________________

## Section III: General Information

1. **Choose all that apply:** *(* See indicated section in HIC Guidelines for Investigators)*

| Children/minors***** (Section E.1) | Pregnant women/fetuses/placenta |
| --- | --- |
| Decisionally impaired***** (Section E.2) | Prisoners |
| Females of childbearing potential  Radioactive Materials  IND # ______________  IDE # ______________A or B (Section C.1) | Non-English Speaking  Use of Employees  Students |

1. **Location of study:** Please identify the hospital, in-patient or outpatient facility, school or other agency that will serve as the location of the research.

Yale University  Yale-New Haven Hospital  APT Foundation, Inc.  West Haven VA

Community Consultation Board, Inc.  Haskins Laboratories  John B. Pierce Laboratory, Inc.

Connecticut Mental Health Center  Hill Health Corporation  Comprehensive Cancer Center

General Clinical Research Center(s)  MR-TAC

Other locations, **Specify**: Connecticut Department of Correction, University of Connecticut

Correctional Managed Health Care

Please indicate the location(s) within the hospital and/or Medical School where the research will take place:

Yale University AIDS Program, 135 College Street, Suite 323, and at

Off Campus Locations: – New Haven Community Correctional Center (New Haven, CT);

York Correctional Institution (Niantic, CT)

*Please note: when other institutions are engaged in the research, it may be necessary to secure the approval of their Institutional Review Boards (IRB) and/or to insure that the institution has obtained a Federal Wide Assurance (FWA). Institutions may not list the Yale HIC as their IRB of record unless the Federal government has approved their FWA and they have in place a fully executed IRB Authorization Agreement between their institution and Yale University.*

1. **Probable Duration of Project**: Please state the expected duration of the project, including all follow-up and data analysis activities. *[Please answer this and all other questions on the form with font size 12.]*

5 Months: (8/01/07 to 12/31/07)

1. **Number of Subjects:** Please state the number of subjects to be enrolled at Yale. For multi-center studies, indicate the total number of subjects to be enrolled across all sites. If different subject populations will participate, state the anticipated number in each group.

A total of ~600 subjects will be recruited. We anticipate 300 subjects will be enrolled at the New Haven Community Correctional Center (NHCCC) and 300 subjects will be enrolled at the York Correctional Institution.

## Section IV: Research Plan

1. **Statement of Purpose:** What are the scientific aims of the study, or the hypotheses to be tested?

The aims of this study are:

1. To simulate and explore the feasibility of future implementation of a routine opt-out HIV testing protocol using rapid testing methods in the often-chaotic jail setting;
2. To determine the ability of subjects to pass a competency assessment in order to consent to routine HIV testing at varying times after entry into jail
3. To examine the acceptability among jail inmates of a routine HIV testing protocol using rapid HIV testing methods;
4. To evaluate the optimal time to perform routine HIV testing among inmates who are

competent to consent to testing, in a manner that not only maximizes public health but also attends to the safety and health status of individual inmates.

1. To assess the attitudes toward future widespread implementation of a routine rapid

HIV testing protocol by correctional, medical, and custodial staff

1. To assess the feasibility of implementing routine HIV testing in jails at different time points during and after entry processing from a systems operations perspective

Hypotheses:

1. A higher proportion of inmates approached for testing the night of intake will be

deemed incompetent to consent to testing (using the standardized MacArthur Competence Assessment Tool )compared to those inmates approached in the days after intake.

1. A higher proportion of inmates approached for testing the night of intake will present at

a higher risk for suicidal ideation and psychological distress (according to the nursing

staff’s mental health assessments and the standardized K6 psychological distress

scale) compared to those inmates approached in the days after intake.

1. A higher proportion of inmates approached for testing the night of intake will not

receive HIV testing (because they are either deemed incompetent or choose not to

provide consent for testing) compared to inmates approached on subsequent days.

1. A significant portion of individuals assigned to being approached for HIV testing on

Day 7 will leave the facility prior to Day 7 and will miss the opportunity to be tested.

1. Correctional medical and custodial staff will express resistance to implementing

widespread routine HIV testing in jail settings.

2. **Background:** Describe the background information that led to the plan for this project. Please provide references to support the expectation of obtaining useful scientific data. When available, previous work in animal and/or human studies should be included.

A number of unique opportunities and challenges exist with regard to HIV testing in jail settings.^1^ The CDC’s recent recommendations to implement routine HIV testing in all healthcare settings acknowledges that HIV testing guided by risk factor assessment alone misses many of those who are HIV-infected.^2^ In 2007, however, HIV testing practices continue to be largely based on the pretesting probability that a patient has identifiable risk factors.^3^

One-quarter of HIV-infected individuals in the United States pass through a correctional facility every year and it is believed that anywhere from one third to one half of these persons are unaware of their HIV status.^4-6^ Because prisons and jails house a population facing a disproportionate share of the HIV infection burden, these facilities serve as important sites for the testing and treatment of HIV.^7^ The prevalence of HIV is four times greater in correctional settings compared to the general population.^8^ Yet, approximately 50% of individuals who enter correctional settings do not know their HIV status and many with HIV infection do not have traditional risk-based behaviors for HIV.^9, 10^ Therefore, enhanced HIV testing strategies in correctional settings remain a high priority. Jails interact with a larger number of individuals at risk for HIV infection than do prisons; however, jails also pose unique logistical and health-related constraints for implementing widespread HIV screening programs. Jails differ from prisons in several important ways that impact HIV testing strategies: 1) jail populations have short periods of incarceration and high rates of turnover with many-fold greater admissions and discharges; 2) jails house individuals with higher rates of acute intoxication from psychoactive drugs, uncontrolled mental illness, and suicidal behavior;^11^ and 3) the individuals who enter jails have higher recent risk behaviors for HIV than those in prisons.^12, 13^ Given these considerations, jails have been less well-equipped to implement screening, prevention, and treatment programs to address patients with or at risk for HIV/AIDS.

Historically, performing HIV testing in jails has been difficult due to brief lengths of incarceration and high turnover of the inmate population,^14^ thereby complicating delivery of test results. Furthermore, jails house individuals whose ability to fully consent to testing is limited due to the high rates of acute intoxication, varying degrees of withdrawal from mind-altering substances and high rates of uncontrolled mental illness of individuals entering this setting. The development of the OraQuick rapid HIV-1 antibody test, however, has created promising opportunities for screening in jail settings, allowing for the prompt delivery of preliminary positive and definitively negative results within twenty minutes.^15^ In addition, revised recommendations by the Centers for Disease Control and Prevention now call for widespread, routine opt-out HIV testing in all health care settings.^2^ Recent cost-benefit analyses also support expanded HIV screening in all settings, especially in sites where the prevalence of HIV exceeds 1%;^16, 17^ the prevalence of HIV in jails in the Northeast exceeds that amount several-fold and would likely yield the highest number of unidentified HIV-infected individuals.

The revised CDC recommendations call for routine testing in all clinical settings, the central goal being to maximize the number of persons who are aware of their HIV infection and receive care and prevention services. According to the CDC guidelines for routine testing, “HIV-negative results may be conveyed without direct personal contact between the patient and the health-care provider.” Only HIV positive results should be communicated confidentially through personal contact by a clinician, nurse, mid-level practitioner, counselor, or other skilled staff. Furthermore, the guidelines state that prevention counseling should not be required as a part of HIV screening programs in health-care settings. ^2^

Understanding the issues related to implementing routine HIV testing using rapid HIV testing methods is challenging yet important for guiding best public health practices in jail settings. The CDC has identified several major issues that must be addressed when developing model routine HIV testing strategies in jails. These include: 1) **choosing the timing of testing after jail entry**; 2) marketing and advertising of services; 3) manner in which testing is presented to inmates; 4) testing protocols; 5) methods of informing those tested of their results; 6) confidentiality of results; 7) HIV counseling associated with testing; and 8) administrative and implementation issues.^18^ From our experience and review of the literature, we also believe that there must also be adequate **screening for and exclusion of individuals from HIV testing who are incompetent to consent (either due to acute intoxication, symptoms of withdrawal, uncontrolled mental illness and/or severe psychological distress) and therefore incapable of “opting out”**.^19^

Current HIV testing practices in U.S. jails are highly variable. Nearly one-fifth of jails report no official HIV testing protocol, ^1^ and as few as 21.6% of jail inmates were HIV tested after admission.^20^ Surveys conducted by the CDC and the National Institute of Justice, report that only 4% of jails provide HIV testing upon inmate request and only 2% offer routine HIV testing on admission; none of those surveyed mandated HIV testing of incoming inmates.^21^

Experience with rapid HIV testing in jail settings is limited.^22, 23^ To date, only two such studies have been published. One study, conducted among female inmates during intake at the Cook County Jail, consisted of approaching detainees in the corner of a large room, in open view of security officers and fellow inmates, and offering them rapid HIV testing. Among this group, 30% were found to be ineligible; however, ineligibility was not systematically defined. Additionally, and perhaps as a result of requiring inmates to consent in a public setting, less than half of those approached were willing to accept testing.^22^ A more recent study, published earlier this year, was conducted at the Rhode Island Department of Corrections Jail, a facility where routine HIV testing has been in place for over 15 years.^23^ Although 95% of participants accepted rapid HIV testing, 87% reported they had been previously HIV tested at this facility during a prior incarceration. Moreover, the time after incarceration was not provided, suggesting that inmates may have been longer term inmates and we were not told how many were ineligible for HIV testing at all. Given these circumstances, concerns remain whether the results of the Rhode Island study can be generalized to other correctional settings. In addition to these studies, the CDC has funded four Jail Demonstration Projects in Florida, Louisiana, New York and Wisconsin, encompassing more than 23 jails in those states. The program, which offers voluntary rapid HIV testing based on referral, has conducted over 33,000 tests and found that 1.1% of individuals were positive, 70% of whom were previously unknown cases of HIV.^24^

None of the aforementioned studies adequately addresses the screening of inmates for psychological distress, intoxication, and withdrawal prior to conducting widespread HIV testing. A major challenge to implementing a routine HIV testing protocol in jails is determining the optimal time to test, taking into account an inmate’s ability to consent to testing and his or her level of psychological distress. Because the incarcerated experience high rates of suicidal behavior, mental illness, acute intoxication, and withdrawal, they might not be competent to consent to HIV testing or to receive a positive HIV test result, even though these same individuals might also be at highest risk for infection.^1, 23^ Therefore, when choosing the appropriate time to screen inmates for HIV, testing must be performed in a manner that not only maximizes public health, but also attends to the safety and health status of individual inmates. It is well documented that inmates are more likely to experience mental distress and present at a higher risk for committing suicide within the first 48 hours of incarceration.^25, 26^ A ten-year study of deaths in the Chicago Cook County jail found that suicides were the third leading cause of death among inmates, following heart and cerebrovascular disease.^27^ In addition, studies of jail populations at time of admission show that acute symptoms of serious mental illnesses requiring treatment are present in about 6% of males and 15% of females at booking.^28, 29^

Prisoners might be too distressed at their time of admission to consider the consequences of being HIV tested. In particular, distressed individuals might not be prepared to receive a positive HIV test result. Furthermore, recently incarcerated prisoners might be intoxicated or experiencing symptoms of withdrawal, thereby preventing them from providing informed consent. The challenge with postponing testing is that many individuals experience very short stays in jail, with about 1/3 leaving within 48 hours, followed by further attrition daily for the first week. Therefore, every day that testing is delayed a greater number of inmates will leave jail without being tested. Although several authors have acknowledged the importance of screening jail inmates for mental status and suicide risk before conducting widespread HIV testing, no literature exists evaluating the tradeoffs between early versus late testing with regard to individual health needs and public health benefit.

**3. Research Plan:** Please provide an orderly scientific description of the study design and research procedures as they

directly affect the subjects.

3.A Prospective Pilot Routine Opt-Out Rapid HIV Testing Study

3.A.1 Overview: This pilot study proposes to perform routine opt-out testing as part of intake for approximately three hundred individuals at each study site (see below for description of study sites). Currently, both of the facilities where this study will be conducted perform voluntary HIV testing upon request. We will work closely with HIV testing counselors, correctional officers, and medical personnel involved with intake procedures in order to simulate as closely as possible future implementation of a routine testing protocol based on current CDC recommendations, using rapid HIV testing methods .

Our primary outcome is the percentage of subjects who consent to and receive an HIV test at a given time-point at each facility. This primary outcome will be assessed independently for each the male and female jails, since the relevant policy question is within each gender rather than between them i.e., policy-making is largely made independently for male and female jails. Additionally, women and men may have different attitudes and feelings toward HIV testing in general, toward HIV testing in jail, and are likely to present with different rates of mental illness, substance withdrawal, etc. that could potentially affect the primary outcome.^30, 31^

Intake procedures are conducted for new arrivals at each facility every Monday through Friday. Those arrested or booked on Friday evening, Saturday, or Sunday must wait in their local court holding facility until the following Monday night when they arrive at York C.I. (females) or New Haven Community Correctional Center (males). In addition, physical exams are conducted for all new arrivals the evening after they arrive at the facility, during second shift (3pm-11pm), i.e. every Tuesday through Saturday.

Inmates who arrive on each intake night (Monday through Friday) will be assigned to be offered testing and approached for participation in the study at one of three time points based on the total number of inmates proceeding through intake that night and their arrival times to the jail. One third of inmates will be assigned to be approached for routine HIV testing and study participation the night of intake itself during routine health screening and mental health assessment (9pm – 1am). One third of inmates will be assigned to be approached for routine HIV testing and study participation the evening following intake when all arriving inmates undergo a routine physical exam. The final third of inmates will be assigned to be approached for routine HIV testing and study participation seven days after arriving at the facility. This last group will not have HIV testing conducted during any routine health procedures as with the first two groups. Even though there are no routine health procedures conducted at this time point, we are still interested in testing this group seven days after intake because many in this testing group would have received seven days of medications and have completed a detox protocol if necessary. We therefore believe that a higher proportion of individuals in this last testing group will be more stable and be competent to consent to testing compared to the prior testing groups, although many of them might have bonded out or been released from the facility by this point. Although this time point is of less interest than the first two time points, because this is a pilot study, we believe that the data we collect from testing at this time point would provide us with valuable information that could better inform our research design for conducting a larger testing study in the future.

3.A.2 Description of Study Sites: We will conduct our study at two jail sites: New Haven Community Correctional Center (men) and the York Correctional Institution (women) located in Niantic. There are on average 25 unique intakes at NHCCC and 20 at York each evening. Services available at both facilities include: a general medical clinic, HIV consultation services, voluntary HIV testing, detox and treatment for those with opioid dependence, standardized detox procedures for alcohol and benzodiazepine withdrawal, an inpatient medical infirmary, a mental health assessment unit, substance abuse counseling, and a variety of support groups. York provides its inmates with methadone treatment for opioid dependence. All inmates undergo a standard, half-day orientation session by a correctional officer the morning after admission to the facility; a health services staff member also provides an overview of services available, including access to voluntary HIV testing. Voluntary HIV testing is available at both sites and can be accessed through inmate request or referral from a clinic based on identified risks or the presence of symptoms associated with HIV. New entrants to the facilities undergo routine medical and psychiatric assessment each weekday evening during the second (3pm-11pm) or third (11pm–7am) shift as part of intake, depending on when they arrive, and are treated according to evidence-based protocols for management of acute intoxication, withdrawal or serious mental illness.

3.A.3 Inclusion/Exclusion Criteria: All newly incarcerated inmates will be eligible.

3.A.4 Sample Size Determination

As stated previously, our primary outcome is the percentage of subjects who consent to and receive an HIV test at a given time-point at each jail, with this primary outcome being assessed independently for each the male and the female jail. The sample size calculations for each jail are exactly the same, even though programmatically relevant and likely observed results and effect sizes are different for each jail. Namely, women are expected to exhibit higher uptake rates.

For both jails, sample size calculations are made comparing the uptake at intake compared to subsequent timepoints. While, for the purposes of this pilot study, we do have three arms to have a more complete programmatic experience, the most relevant analysis is to just compare intake with post-intake. Since we are randomizing 1:1:1, this results in a 2:1 comparison for the primary outcome comparing the intake group with the collapsed post-intake groups. Table 1 provides sample size estimates for each arm, based on using the chi-square tests to compare the proportion uptake at each time-point within each jail setting and calculated using the standard sample size formula:

Where p is the proportion who receive an HIV test at intake, alpha is the type 1 error rate, beta is the type 2 error rate, and delta is the difference between the intake and post-intake groups. Plausible ranges of uptake rates are garnered from our current experiences in the jail system, through extensive clinical interviews and discussions with jail counselors. Only one other study has assessed routine testing in a jail setting. That study, however, is likely to have significantly higher uptake rates than will be seen in our study, since many of the subjects were repeat testers and the program had been running in that jail for some time.^23^

At our best-estimated initial uptake of 60% for men and 75% for women, 100 subjects in each arm will detect differences of 16% and 13%, respectively, with a type 1 error rate of 0.05 and power of 80%.

| **Table 1. Sample Size Calculations** | | |  |  |
| --- | --- | --- | --- | --- |
| **Jail Setting** | **Intake** | **Subsequent Time-points** | **N (each arm)** | **3N (Total Subjects)** |
| Men | 50% | 65% | 126 | 378 |
|  | 55% | 70% | 121 | 363 |
|  | **60%** | **76%** | **98** | **294** |
|  | 65% | 80% | 101 | 303 |
|  | 70% | 84% | 103 | 309 |
| Women | 65% | 80% | 101 | 303 |
|  | 70% | 84% | 103 | 309 |
|  | **75%** | **88%** | **100** | **300** |
|  | 80% | 92% | 93 | 279 |
|  | 85% | 95% | 99 | 297 |

3.A.5 Procedures

3.A.5.1 Testing

1. Inmates who arrive on each intake night (Monday through Friday) will be assigned to be offered testing and approached for participation in the study at one of three time points based on the total number of inmates proceeding through intake that night and their arrival times to the jail. One third of inmates will be assigned to be approached for routine HIV testing and study participation the night of intake itself during routine health screening and mental health assessment (9pm – 1am). One third of inmates will be assigned to be approached for routine HIV testing and study participation the evening following intake when all arriving inmates undergo a routine physical exam. The final third of inmates will be assigned to be approached for routine HIV testing and study participation seven days after arriving at the facility.
2. Depending on the testing group to which the inmate is assigned (Day 0, Day 1, or Day 7 – see figure below), either the intake nurse, the physician’s assistant performing physical exams, or a member of the research study staff will offer to swab the inmate for HIV testing. It will be explained that HIV testing is now done using an oral swab and that results can be made available within a short period of time. Individuals offered testing on Day 0 (intake night) will be told that they will receive their results the following morning. Individuals tested on Day 1 and Day 7 will be told they will receive their results that afternoon/evening. The inmate will be instructed that HIV testing, like many other conditions, can be performed as part of routine screening in the jail setting, similar to other medical conditions such as substance abuse urine testing, and screening for tuberculosis, pregnancy (for women) and hypertension.
3. At this point, the inmate will be given an opportunity to opt-out of being swabbed for testing. If the inmate opts out and is part of the Day 0 or Day 1 testing group, s/he will be invited back for testing on Day 1 or Day 7, respectively. If the inmate is part of the Day 7 testing group, s/he will not be approached again for testing.
4. If the inmate agrees to be swabbed, the inmate will meet with a member of the research study staff who will ask the subject to provide 2 separate written consents – one to participate in the study, and one to have the swab tested for HIV. Only research staff will be obtaining consent for this study.
5. At this point, on Day 0 and Day 1, the inmate will be given another opportunity to opt-out of consenting to participate in the study and to have the swab HIV tested. If the inmate opts out and is part of the Day 0 or Day 1 testing group, s/he will be invited back for testing on Day 1 or Day 7, respectively. If the inmate is part of the Day 7 testing group, s/he will not be approached again for testing.
6. If the inmate provides written consent, s/he will be administered the standardized MacArthur Competency Assessment Tool by a member of the study staff. The competency assessment tool will determine if the inmate understands the purpose of HIV testing and the potential benefits and risks or discomforts of choosing to test.
7. If the staff member deems the inmate competent according to the standardized tool, the inmate’s swab will be tested for HIV and the inmate will participate in an interview. The interview will include standardized instruments to assess opiate and alcohol withdrawal, psychological distress, and medical ability to consent to treatment. The schedule for administering the questionnaire is detailed in Table 1 below. The inmate’s swab will be tested immediately. Testing will be performed by the research study staff. Results will be recorded, and the swab will then be discarded in a hazardous waste container.
8. If the inmate is deemed not competent according to the standardized tool, further interviewing will be halted. The swab will be immediately discarded in a hazardous waste container and will not be tested. The inmate will be informed that even though s/he has consented to be HIV tested, the study staff does not believe that now is a good time for him or her to be HIV tested. If the inmate is part of the Day 0 or Day 1 testing group, the study staff will inform the inmate that he or she will be invited back for testing in the near future. Inmates in all three testing groups will also be informed that if this is not satisfactory, then they can always request an HIV test from the HIV testing counselors.
9. To explore feasibililty among correctional medical and custodial staff members, we will conduct one on one interviews with nurses, HIV counselors, mental health personnel, drug treatment staff, health services administrative staff members, and custodial staff to explore their attitudes about routine HIV testing. We will approach those staff members involved with intake procedures as well as medical staff.

Table 1: Schedule of Interviews

| **Interview Instrument** | **Administered to Inmate** | **Administered to Staff** |
| --- | --- | --- |
| MacArthur Competence Assessment to evaluate medical ability to consent to testing | X |  |
| Demographics | X |  |
| History of previous HIV testing experiences | X |  |
| Court dates | X |  |
| Likelihood of remaining incarcerated | X |  |
| Willingness to HIV test now if it were made available | X |  |
| Attitudes about HIV testing in jails and prisons | X |  |
| Clinical Opiate Withdrawal Scale (COWS) | X |  |
| Clinical Institute Withdrawal Assessment of Alcohol (CIWA) | X |  |
| National Center for Health Statistics (NCHS) K6 Scale for Serious Psychological Distress | X |  |
| Staff attitudes about routine HIV testing in jails |  | X |

3.A.5.2 Delivery of HIV Test Results

1. Timing of delivery of test results will differ depending on the testing group. For the Day 1 and Day 7 testing groups, delivery of test results will be given the same evening that testing occurs. For the Day 0 testing group, however, delivery of test results will be given the following morning instead of the night of intake. Although this protocol is to determine the feasibility of future implementation of a rapid routine HIV testing program, for the purposes of this study it is not logistically possible for us to deliver preliminary positive test results the night of intake. This is because delivery of preliminary positive test results must be done by the DOC HIV counselors. Because intake procedures only start around 9pm and continue very late into the night, it is not logistically possible for us to hire the DOC HIV testing counselors to work during those hours, as they must work the first shift (7am-3pm). Therefore, after extensive consultation with jail HIV testing counselors, nursing and medical staff, and Brian Goodrich, the HIV Prevention Program Manager for the Department of Corrections, we have decided to have delivery of all test results for the Day 0 testing group delivered early the following morning starting at 6:30AM. The counselors have agreed to be on call to come back during the 2^nd^ shift (3pm-11pm) to deliver positive results to inmates tested on Days 1 and 7. For each facility, we have made these arrangements with HIV counselors who live in close geographic proximity to each jail.
2. Delivery of all preliminary positive results will be given by HIV testing counselors working at both correctional facilities. Delivery of all negative results, however, will be delivered by research study staff. One of our main reasons for choosing this method is because we want to ensure that subjects diagnosed as preliminarily positive have sufficient time to meet with counselors for post-test counseling. Also, if counselors must meet with inmates to deliver both positive and negative results, they may not have enough time to deliver all results prior to new inmates needing to return to court or bonding out. This would mean that some individuals in the Day 0 testing group could bond out or be taken to court in the morning before receiving test results. The bondsman arrives at 8am at both facilities – therefore, in order to ensure that all subjects receive their test results before meeting with the bondsman, both positive and negative test results will be delivered early the following morning starting at 6:30AM.
3. Although delivery of negative test results by research study staff is not the current standard of care at both study sites, it does simulate the standard of care that would be delivered if routine HIV testing were to be adopted. According to the revised CDC guidelines for routine testing, “HIV-negative results may be conveyed without direct personal contact between the patient and the health-care provider.” Only HIV positive results should be communicated confidentially through personal contact by a clinician, nurse, mid-level practitioner, counselor, or other skilled staff. Furthermore, prevention counseling should not be required as a part of HIV screening programs in health-care settings. ^2^ This protocol of delivery of negative test results by research study staff has also been reviewed and preliminarily approved by the Department of Corrections Research Committee, pending a signature from the Deputy Commissioner. The DOC Research Committee must give final approval to any study proposed to be conducted in the Connecticut Department of Corrections.
4. The research staff working on this study have considerable collective experience with HIV testing, counseling, as well as interacting with inmates and providing healthcare to the incarcerated. One member of the study staff has been a prison nurse with the Department of Corrections for over twenty years, and has been involved with providing intake health assessments at York C.I. for over ten years. Another member of the team is a Connecticut Department of Public Health certified HIV counselor and has many years of experience conducting rapid HIV testing and counseling. All members of this team have had experience conducting rapid HIV testing and delivering negative test results.
5. Although we are using rapid HIV tests for this study, we will inform the inmates approached for the study that test results can be received “within a short period of time”, rather than specifying that results can be received in as little as 20 minutes. The reason we have chosen to do this is because we do not want to create an expectation that if the inmate chooses to be tested, s/he will receive results in 20 minutes. As we stated above, although this study aims to simulate future implementation of a routine rapid HIV testing protocol, there are operational and logistic challenges to delivering all results in twenty minutes both for this study and in true clinical practice. If, for instance, there is a preliminary positive test result in the Day 0 testing group, we will have to wait until the morning in order for the counselor to deliver the result. For the Day 1 and Day 7 testing groups, we will have to call the HIV counselor to come in to deliver a preliminary positive test result, which will likely take more than twenty minutes. We do not want to create additional anxiety for the inmates by exposing them to a longer than expected waiting period for results. If routine testing were actually implemented in this setting, there are a number of factors, such as a medical or behavioral emergency with another inmate that could preclude delivery of test results within twenty minutes.

3.A.6 Study Design (see figure below)

INMATES ARRIVE AT YORK C.I. OR NEW HAVEN C.C.C. FOR INTAKE PROCEDURES

**DAY 0:** 1/3 TESTED ON INTAKE NIGHT DURING HEALTH ASSSESSMENT

**9PM – 2AM**

**MONDAY-FRIDAY**

**DAY 1:** 1/3 TESTED EVENING AFTER INTAKE DURING PHYSICAL EXAM

**5PM -9PM**

**TUESDAY-SATURDAY**

**DAY 7:** 1/3 TESTED 7 DAYS AFTER INTAKE NIGHT

**5PM -9PM**

**MONDAY-FRIDAY**

**PHYSICIAN’S ASSISTANT** OFFERS TO SWAB INMATE FOR HIV TESTING AS PART OF ROUTINE CARE

**RESEARCH STUDY STAFF** OFFERS TO SWAB INMATE FOR HIV TESTING AS PART OF ROUTINE CARE

**INTAKE NURSE** OFFERS TO SWAB INMATE FOR HIV TESTING AS PART OF ROUTINE CARE

DOES NOT CONSENT

CONSENTS

DOES NOT CONSENT

CONSENTS

CONSENTS

RESEARCH STUDY STAFF CONSENTS SUBJECT FOR STUDY AND HIV TEST

RESEARCH STUDY STAFF CONSENTS SUBJECT FOR STUDY AND HIV TEST

RESEARCH STUDY STAFF CONSENTS SUBJECT FOR STUDY AND HIV TEST

COMPETENT

INTERVIEWED, SWAB TESTED

NO INTERVIEW, NO SWAB TESTED

INTERVIEWED, SWAB TESTED

NO INTERVIEW, NO SWAB TESTED

INTERVIEWED, SWAB TESTED

INCOMPETENT

COMPETENT

INCOMPETENT

COMPETENT

INCOMPETENT

RESULTS GIVEN **SAME EVENING**

RESULTS GIVEN **SAME EVENING**

NOT INVITED BACK FOR TESTING

INVITED BACK FOR **DAY 7** TESTING

RESULTS GIVEN **NEXT** **MORNING**

RESEARCH STUDY STAFF PERFORMS COMPETENCY ASSESSMENT

RESEARCH STUDY STAFF PERFORMS COMPETENCY ASSESSMENT

RESEARCH STUDY STAFF PERFORMS COMPETENCY ASSESSMENT

DOES NOT CONSENT

NO INTERVIEW, NO SWAB TESTED

INVITED BACK FOR **DAY 1** TESTING

INMATE OPTS OUT

INMATE SWABBED

INMATE OPTS OUT

INMATE SWABBED

INMATE OPTS OUT

INMATE SWABBED

3.A.7 Analytic Plan

Ineligibility for routine HIV testing will be defined as those inmates who are medically incompetent using the MacArthur Competence Assessment Tool or who are otherwise found to be suicidal or in need of psychiatric holding by DOC staff. To address the first hypothesis, we will compare the proportion of individuals who are ineligible in each testing group using Mantel Haenszel testing. Because we anticipate that as many as 50% of jail detainees will be released within the first 72 hours, we will compare the proportion who accept testing in each group using Mantel Haenszel testing. Last, we will conduct multiple logistic regression of all intakes to assess the correlates of accepting mouth swabs as well as the correlates of accepting HIV testing. Secondary variables of interest will include demographic characteristics (it is possible, for example, that non-whites may be less likely to accept testing), drug use characteristics, presence of psychological distress, addiction severity scores, and history of past HIV testing. Data from this component of the study will be used to determine the effect size of a larger randomized controlled trial of routine HIV testing in jails.

## Section V: Research Involving Drugs, Devices or Biologics

Please note: protocols using chemicals, hormones, other natural substances, or devices not regulated by the U.S. Food and Drug Administration (FDA) must still complete this section of the application form. Based upon the information provided in items 1-4, the HIC will determine whether an Investigational New Drug (IND) or Investigational Device Exemption (IDE) application must be submitted to the FDA.

1. **Identification of Drug, Device or Biologic:** What is the name of the drug, device or biologic being used? Please identify whether FDA approval has been granted, and for what indication(s).
2. **Background Information:** Please provide a description of previous human use, known risks, and data addressing dosages, intervals, routes of administration, and any other factors that might influence risks.
3. **Source:** Please identify the source of the drug, device or biologic to be used.
4. **Preparation and Use:** Please describe the method of preparation, storage, stability information, and for parenteral products, method of sterilization and method of testing sterility and pyrogenicity.
5. **Use of an Investigational Drug, Device or Biologic:** Protocols which utilize a drug, device or biologic not approved by the FDA must provide the following information.

- What is the Investigational New Drug (IND) or Investigational Device Exemption (IDE) number assigned by FDA?
- For IDE’s: Did the FDA approve this IDE as a Category A (experimental/investigational) or as a Category B (non-experimental/investigational)?
- Who holds the IND or IDE?
- Is the drug or device provided free of charge by the Sponsor? Yes ___ No ___

1. The FDA requirements to qualify for an exemption from filing an IND are as follows [21 CFR 312.2(b)]:

Exemptions – The clinical investigation of a drug product that is lawfully marketed in the United States is exempt from the requirements filing for an IND if ALL the following apply:

- - 1. The investigation is not intended to be reported to FDA as a well controlled study in support of a new indication for use nor intended to be used to support any other significant change in the labeling for the drug;
    2. If the drug that is undergoing investigation is lawfully marketed as a prescription drug product, the investigation is not intended to support a significant change in the advertising for the product;
    3. The investigation does not involve a route of administration or dosage level or use in populations or other factor that significantly increases the risks (or decreases the acceptability of the risks) associated with the use of the drug product;
    4. The investigation is conducted in compliance with the requirements for institutional (HIC) review and with the requirements for informed consent of the FDA regulations (21 CFR Part 50 and 21 CFR Part 56);
    5. The investigation is conducted in compliance with the requirements regarding promotion and charging for investigational drugs and
    6. The investigation is not emergency research (21 CFR Part 50.24).

1. Use of Placebo: Does any part of the study involve placebo? Yes ___ No ___
   1. If yes, please address each one of the following:
      1. The safety and efficacy of other available therapies (if any).
      2. The maximum total length of time a participant may receive placebo on study.
      3. The greatest potential harm that may come to a participant as a result of not receiving effective therapy (immediate or delayed onset).
      4. Protocols in place to safeguard participants receiving placebo.

## Section VI: Human Subjects

1. **Recruitment Procedures:** How will potential subjects be identified, contacted and recruited? Attach copies of any recruitment materials – such as flyers, telephone scripts, or introductory letters – that will be used.

Please note that a researcher may not use an individual’s Protected Health Information (PHI) for recruitment into research without first obtaining an authorization from the individual, or a Waiver of Authorization from the HIC. A treating provider does, however, have the option to:

- Discuss with his/her own patients the option of enrolling in a study.
- Obtain written authorization from the patient for referral into a research study.
- Provide background information about the study to the patient so that the patient can initiate contact with the researcher.
- Provide the individual’s PHI to a researcher without authorization when the researcher has obtained an approved Waiver of Authorization for recruitment purposes from the HIC.

If PHI will be accessed without subject authorization, please state whether any member of the research team has an existing clinical relationship with the potential subject. Researcher-clinicians are permitted to access the PHI of their own patients, or patients of co-investigators listed on the protocol, for recruitment purposes. Alternately, please submit a completed Yale University Request for HIPAA Waiver of Authorization for Research Form (available at <http://info.med.yale.edu/hic/hipaa/>. *For further information, see the Yale HIPAA website at* <http://info.med.yale.edu/hipaa/>.

New entrants to the facilities undergo routine medical and psychiatric assessment each evening during the second (3pm-11pm) and third (11pm–7am) shift and are treated according to evidence-based protocols for management of acute intoxication, withdrawal or serious mental illness. They also undergo a physical exam the evening after intake processing. Recruitment for this study will take place during intake procedures, during the physical exam that happens the day after intake, and 7 days after intake during the second shift.

Inmates who arrive on each intake night (Monday through Friday) will be assigned to one of three testing groups based on the total number of inmates proceeding through intake that night and their arrival times to the jail. One third of inmates will be assigned to be approached for routine HIV testing and study participation the night of intake itself during routine health screening and mental health assessment (9pm – 1am). One third of inmates will be assigned to be approached for routine HIV testing and study participation the evening following intake when all arriving inmates undergo a routine physical exam. The final third of inmates will be assigned to be approached for routine HIV testing and study participation seven days after arriving at the facility. These procedures have been described above in the previous section.

1. **Inclusion/Exclusion Criteria:** What are the criteria for subject inclusion or exclusion? How will eligibility be determined, and by whom?

All newly incarcerated inmates will be eligible to be approached for testing and inclusion in this study. Individuals who are suicidal or incompetent to consent will be excluded from the study. We will keep track of how many people are incompetent to consent, but we will not keep data on those individuals or carry out the testing. The standardized MacArthur Competence Assessment, as well as the clinical judgment of the DOC clinicians who put individuals on suicide watch or psychiatric holds will be used to determine whether or not someone is eligible to participate.

1. **Subject Population:** Provide a detailed description of the proposed involvement of human subjects. Describe the characteristics of the subject population, including their anticipated number, age range and health status.

#### We will recruit a total of 600 newly incarcerated jail inmates during intake orientation between both facilities. For an explanation of sample size requirements, please see the Sample Size subsection under heading IV. Research Plan above.

New Haven Correctional Center houses only males; the population at York C.I. consists of only females. Therefore, as indicated in the gender and minority inclusion statement, women will be equally represented in recruitment since the study will be recruiting an equal number of men and women from both sites. Minorities will also be consistently represented in both sites of recruitment. Given the demographics of Connecticut jails, it is estimated that roughly 80% of study participants will be Black and/or Hispanic, reflective of the inmate population overall. The minimum age for participation is 18 years of age; thus children 18-21 years old will be eligible for participation.

The selection of subjects should be equitable. Generally speaking, the subject selection should reflect a reasonable cross-section of the population that is being studied. In research that requires a more restricted population, the rationale for this need should be fully justified. Investigators must also provide scientific justification for the exclusion of underrepresented populations such as women, children, or minorities.

1. **Vulnerable Subjects:** Certain populations are considered to be vulnerable and require special protections when asked to participate in a research study. *(Vulnerable populations include, but are not limited to pregnant women, fetuses, human embryos, prisoners, children, and cognitively impaired individuals or persons with questionable capacity to consent. Others which may require special consideration include elderly persons, economically disadvantaged persons and educationally disadvantaged persons.)*

*Children include all persons who have not attained the legal age for consent to treatments or procedures involved in the research under the applicable law of the jurisdiction in which the research will be conducted (the age of majority is 18 years in the Connecticut). Parental permission and the child’s assent is required for participation in the study. (Assent is defined as “a child’s affirmative agreement to participate in research” and should be sought in addition to parental permission when the minor subject is sufficiently mature to understand the nature of his or her participation in a research study.) Please refer to the HIC Guidelines for Investigators at* [*http://www.med.yale.edu/hic/forms/forms/guidelines.pdf*](http://www.med.yale.edu/hic/forms/forms/guidelines.pdf)

Will vulnerable subjects be enrolled in the study? If so, identify the vulnerable population and provide a justification for their involvement. Also address any additional safeguards necessary to protect the rights and welfare of vulnerable subjects.

All subjects recruited into this study will be vulnerable because of their recent incarceration status. The rationale for their inclusion as vulnerable subjects is that inmates not only face a disproportionate share of the HIV infection burden, but they also experience comparably high rates of suicidal behavior, mental illness, acute intoxication, and withdrawal. This study is designed to develop an effective routine HIV testing protocol in jails in a manner that not only maximizes public health benefit but also attends to the safety and health status of individual inmates.

Because inmates are often subject to coercion, we intend to protect them in the following ways: 1) offer no incentives to participation while the subject is still a prisoner; and 2) clarify that receiving health care or other services while incarcerated is NOT dependent upon participating in the study. Inmates will also be informed that if they wish to be tested for HIV and don’t wish to participate in the study, that they may request a test from the DOC HIV counselors.

## Section VII: Consent/Assent Procedures

- 1. **Consent Personnel:** Please list all personnel who will be obtaining consent.

Actual consent for participation in this study will be obtained only by study personnel listed on our protocol. No CMHC or DOC personnel will be consenting subjects to participate in this study.

- 1. **Assessment of Capacity to Consent:** For research involving subjects with limited decision-making capacity, how will the capacity to consent be assessed?

The mean level of education of prisoners is completion of an 11^th^ grade education. Many cannot read or write. For this reason, the research staff will read the consent form and inquire about understanding the consent as well answer field questions that the subject may have.

After obtaining consent, research staff will administer the 21 item MacArthur Competence Assessment tool. Any participant who scores medically incompetent to consent will be deemed incapable of consenting for that day, and the consent will be considered invalid. The inmate will not be interviewed further and the test will not be performed. Inmates who are deemed incapable of consenting on Day 0 and Day 1 will be re-approached and re-evaluated on Days 1 and 7 respectively.

- 1. **Process of Consent:** Describe the setting and conditions under which consent will be obtained, including any steps taken to enhance subjects’ independent decision-making.

Inmates who arrive on each intake night (Monday through Friday) will be assigned to one of three testing groups based on the total number of inmates proceeding through intake that night and their arrival times to the jail. One third of inmates will be assigned to be approached for routine HIV testing and study participation the night of intake itself during routine health screening and mental health assessment (9pm – 1am). One third of inmates will be assigned to be approached for routine HIV testing and study participation the evening following intake when all arriving inmates undergo a routine physical exam. The final third of inmates will be assigned to be approached for routine HIV testing and study participation seven days after arriving at the facility. Depending on the testing group to which the inmate is assigned (Day 0, Day 1, or Day 7 – see figure above), either the intake nurse, the physician’s assistant performing physical exams, or a member of the research study staff will offer to swab the inmate for HIV testing. It will be explained that HIV testing is now done using an oral swab and that results can be made available within a short period of time. S/he will instruct the inmate that HIV testing, like many other conditions, can be performed as part of routine screening in the jail setting, similar to other medical conditions such as substance abuse urine testing, and screening for tuberculosis, pregnancy (for women) and hypertension.

At this point, the inmate will be given an opportunity to opt-out of being swabbed for testing. If the inmate opts out and is part of the Day 0 or Day 1 testing group, s/he will be invited back for testing on Day 1 or Day 7, respectively. If the inmate is part of the Day 7 testing group, s/he will not be approached again for testing. If the inmate agrees to be swabbed, after the physical screening is concluded the inmate will meet with a member of the research study staff in a private, screened off area adjacent to the exam room. There the study staff will ask the subject to provide 2 separate written consents – one to participate in the study, and one to have the swab tested for HIV. Only research staff will be obtaining consent for this study.

At this point, the inmate will be given another opportunity to opt-out of consenting to participate in the study and to have the swab HIV tested. If the inmate opts out and is part of the Day 0 or Day 1 testing group, s/he will be invited back for testing on Day 1 or Day 7, respectively. If the inmate is part of the Day 7 testing group, s/he will not be approached again for testing. If the inmate provides written consent, s/he will be administered the standardized MacArthur Competency Assessment Tool by a member of the study staff. The competency assessment tool will determine if the inmate understands the purpose of HIV testing and the potential benefits and risks or discomforts of choosing to test.

If the staff member deems the inmate competent according to the standardized tool, the inmate’s swab will be tested for HIV and the inmate will participate in an interview. The interview will include standardized instruments to assess opiate and alcohol withdrawal, psychological distress, and medical ability to consent to treatment. The schedule for administering the questionnaire is detailed in Table 1 below. The inmates swab will be tested immediately. Testing will be performed by the research study staff. Results will be recorded, and the swab will then be discarded in a hazardous waste container.

If the inmate is deemed not competent according to the standardized tool, further interviewing will be halted. The swab will be immediately discarded in a hazardous waste container and will not be tested. The inmate will be informed that even though s/he has consented to be HIV tested, the study staff does not believe that now is a good time for him or her to be HIV tested. If the inmate is part of the Day 0 or Day 1 testing group, the study staff will inform the inmate that he or she will be invited back for testing in the near future. Inmates in all three testing groups will also be informed that if this is not satisfactory, then they can always request the HIV testing counselors for an HIV test.

- 1. **Non-English-Speaking Subjects:** For research involving non-English-speaking subjects, fully explain provisions in place to ensure comprehension. In addition, please submit translated copies of all consent materials.

The study staff and HIV counselors at both sites include individuals who are bilingual/bicultural in Spanish and English. A Spanish consent form has been submitted for approval. No mono-lingual Spanish speakers will be enrolled until final HIC approval of the Spanish consent form.

- 1. **Parental Permission and Assent:** For research involving minors, please explain how parental permission and child assent will be obtained.

Not applicable

- 1. **Documentation of Consent:** Specify the forms that will be used among the following: adult consent form, parental permission form, LAR (Legally Authorized Representative) permission form, adult assent form, adolescent assent form (ages 13-17 inclusive), child assent form (ages 7-12 inclusive), and information sheet. Copies of all forms should be appended to the protocol, in the same format that they will be given to subjects.

Attached

- 1. **Waiver of Consent:** Will you request either a waiver of consent, or a waiver of signed consent, for this study? If so, please address the following:

We do not request a waiver of consent or a waiver of signed consent for this study.

*Waiver of consent*:

- 1. Does the research pose greater than minimal risk to subjects?
  2. Will the waiver adversely affect subjects’ rights and welfare?
  3. Why would the research be impracticable without the waiver?
  4. How will pertinent information be returned to subjects, if appropriate at a later date?

*Waiver of signed consent*:

1. Does the research pose greater than minimal risk? If so, does a breach of confidentiality constitute the principal risk to subjects?
2. Would the signed consent form be the only record linking the subject and the research?
3. Does the research include any activities that would require signed consent in a non-research context?
   1. **HIPAA Authorization:** If the research involves the creation, use or disclosure of PHI, separate authorization is required under the HIPAA Privacy Rule. Please provide the HIPAA Research Authorization Form and/or a request for waiver of HIPAA authorization. *(For further information, see the Yale HIPAA website at* <http://info.med.yale.edu/hipaa/>).

Attached.

## Section VIII: Protection of Research Subjects

1. **Risks:** What are the reasonably foreseeable risks, discomforts, or inconveniences associated with participation in the research?

The risks in this study are minimal – potential risks could however include breaches of confidentiality within the jail. If jail guards or other inmates were to learn of an individual’s HIV status, it could lead to discriminatory behavior and even physical harm. In addition, learning of one’s own HIV status could be both upsetting news and potentially difficult information for inmates to deal with constructively, especially within the often chaotic correctional setting. Lastly, it is possible that with the high turnover in the jail setting, an inmate might test preliminarily positive using the OraQuick rapid HIV test but will be released before a confirmatory result can be delivered.

The published sensitivity and specificity for the OraQuick Advance Rapid HIV-1/2 Antibody oral test are 99.3% (95% confidence interval [CI] = 98.4% -- 99.7%) and 99.8% (CI = 99.6% -- 99.9%). According to these reported rates, it would be expected that for every 1000 individuals tested, 2 would receive false positive results.

Our research protocol proposes to test 600 individuals. Therefore, while it is still possible for one of our subjects to falsely test positive, the likelihood is very small.

Please note: Potential research risks include more than physical harm; risks may also include, for example, emotional or psychological harm, risk of social stigmatization, economic or legal risk.

1. **Minimizing Risks:** How will the above-mentioned risks be minimized?

Potential instances for breaches of confidentiality include during the interview process or during delivery of positive test results. From our research efforts and provision of clinical care for inmates within the correctional system, we have paid careful attention to potential breaches of confidentiality for subjects undergoing interviews. Dr. Altice, the Principal Investigator, has written extensively on the legal and ethical issues with regard to conducting clinical trials among prisoners.^30,31^ All interviews will be conducted individually during the second shift when rooms are more accessible for consent procedures and privacy can be ensured. Rooms are private and the information collected from individual interviews will be confidential. Appointments will be staggered to avoid participant encounters in the interview room or in the outside hallway. All information will be stored in password-protected computers with double-password protection for opening specified files. All confidential information (study instruments, HIV status, etc.) will be recorded with study participant number only and maintained in locked cabinets within our offices at the Yale University AIDS Program and will only be available to be opened by the Study Coordinator, study staff, or the Principal Investigator. Electronic databases will be maintained through password-protected computers and files and maintained on a single computer at the Yale University AIDS Program. No data will be stored on laptops, flash drives, or be transported via email or any other medium. The data will be stored according to a study ID number and therefore will contain no names, prison ID numbers, or any other personal identifiers. Identifying information will not be stored. All data will be encrypted using encryption software provided by Yale Information and Technology Services.

Study participants should not have to worry about being identified as HIV+ by participating in the study, since all newly entering inmates will be eligible, most of whom will not be HIV+. We have designed this study so that capacity is not so excessive that confidentiality is likely to be breached.

All inmates who test preliminarily positive will receive post-test counseling from trained DOC HIV testing counselors. Any inmate who describes suicidal ideation, or any other acute medical or psychiatric condition, will be immediately referred to the prison health staff for professional assessment. Preliminarily positive patients will then undergo confirmatory testing with either a Western blot or immunofluorescent assay (IFA) for confirmation of test results. Actual consent for participation in this study will be obtained only by study personnel listed on our protocol. No CMHC or DOC personnel will be consenting subjects to participate in this study. All testing and delivery of preliminary positive results will be conducted by these trained counselors in coordination with study staff using existing medical facilities within the jail setting and will be documented appropriately in accordance with DOC confidentiality protections.

Among those inmates who receive HIV testing, the incidence of HIV is estimated to be approximately 2% in correctional settings in the United States. According to Connecticut law, all inmates testing positive for HIV infection are entitled to treatment within the correctional setting. Under these guidelines, the DOC will be expected to pay for treatment of all subjects identified as HIV positive. Administrators from Connecticut Correctional Managed Healthcare (CMHC) have expressed their willingness to participate in this study and are aware of their obligation to treat subjects identified as HIV positive.

Continuity of care is provided for all HIV-positive inmates housed in the Connecticut correctional system through Project TLC (Transitional Linkage to Care).  Project TLC was created by the PI for this protocol and has been in place in the Connecticut Department of Corrections since 1997.  Prior to release, every HIV positive-inmate will meet with a case manager who will conduct a needs assessment for the inmate.  The needs assessment includes:

- a two weeks discharge supply of free antiretroviral medications and other medications the inmate is currently taking
- the case manager completes the necessary paperwork for the inmate to participate in the AIDS Drug Assistance Program upon release, which provides free antiretroviral medication once the inmate is in the community.  Inmates are also strongly advised to apply for reinstatement of SAGA (State-Administered General Assistance), administered by the Connecticut State Dept of Social Services, as well as for Title 19 which provides Medicaid.
- the case manager schedules an appointment for the inmate to meet with an HIV provider in geographical proximity to the inmate’s local community.

1. **Data and Safety Monitoring Plan:** Please include a Data and Safety Monitoring Plan (DSMP) that includes an explicit statement of overall risks, addresses attribution and grading of adverse events and describes procedures for monitoring the ongoing progress of the research and reporting adverse events. For more information, please see the HIC website: <http://info.med.yale.edu/hic/forms/index.html>

Because we are not proposing a clinical trial, it is not appropriate to have a Data and Safety Monitoring Board (DSMB). However it is essential to have a Data and Safety Monitoring Plan (DSMP). We are proposing conducting a prospective observational study to evaluate acceptability of routine testing HIV and a prospective routine HIV testing study in the jail setting. The PI and research staff will review any reports of adverse events related to the study. Serious, unanticipated adverse events will be reported within 48 hours to the HIC, and funding agencies. There are no anticipated serious adverse effects. However, listed below are a number of items that could potentially be adverse events for participation in this study:

- 1. Breaches of confidentiality: Participants will be notified that they should let study staff or the HIV testing counselor know if there are any breaches in confidentiality. If a breach were reported, this would then be reported immediately to the rest of the study team and principal investigator. An initial investigation would be undertaken and simultaneously reported to the HIC using standard university procedures.
  2. Distress due to learning HIV status: Learning one’s HIV status could be both upsetting news and potentially difficult information for inmates to deal with constructively, especially within the often chaotic correctional setting. All inmates who test preliminarily positive will receive post-test counseling from trained jail HIV testing counselors. Any inmate who describes suicidal ideation, or any other acute medical or psychiatric condition, will be immediately referred to the prison health staff for professional assessment. Any instance of an inmate who harms his/herself as a result of learning his/her HIV status will be immediately reported to the HIC.
  3. Confidentiality of research data: Breaches of confidentiality regarding research data are more difficult to detect. For that reason, we have in place a number of safeguards including locked file cabinets and double password-protected computers. Electronic databases will be maintained through password-protected computers and files and maintained on a single computer at the Yale University AIDS Program. No data will be stored on laptops, flash drives, or be transported via email or any other medium. The data will be stored according to a study ID number and therefore will contain no names, prison ID numbers, or any other personal identifiers. Identifying information will not be stored. All data will be encrypted using encryption software provided by Yale Information and Technology Services. In accordance with federal and university policy, all research personnel are compliant with HIPAA training. If any breach of data confidentiality were detected, we would use similarly detailed procedures for report to the PI and HIC simultaneously.

1. **Confidentiality:**
   1. Will private identifiable information about individuals be collected and used?
   2. All confidential information (study instruments, test results, etc.) will be recorded with a study participant number only. This study participant number will be different from the participant’s prison ID number and will in no way serve as a personal identifier. A HIPAA Research Authorization form will be signed by all participants. Information collected will be maintained in locked cabinets within our offices at the Yale University AIDS Program, and will only be available to be opened by the Study Coordinator, study staff, or the Principal Investigator. No data will be stored on laptops, flash drives, or be transported via email or any other medium. The data will be stored according to a study ID number and therefore will contain no names, prison ID numbers, or any other personal identifiers. Identifying information will not be stored. All data will be encrypted using encryption software provided by Yale Information and Technology Services. In accordance with federal and university policy, all research personnel are compliant with HIPAA training. If any breach of data confidentiality were detected, we would use similarly detailed procedures for report to the PI and HIC simultaneously.
   3. How will research data be collected and recorded?

Research data will be collected via interview and via the carrying out of HIV testing after consent as described above.

In order for subjects to be invited back on subsequent days for interviews or for delivery of test results their names and prison ID number must be recorded. These names and prison ID numbers will be recorded on a sheet along with a unique study ID number for each subject. This will be the only record linking study number and subject identification. This sheet will be kept in a locked cabinet in the HIV testing counselor’s office at each study site. Only the HIV testing counselor and study personnel will have access to this sheet. At no time will this sheet leave the HIV testing counselor’s office.

The interview forms, informed consent documents, and the swabs used for the rapid HIV tests will be labeled only with the study ID number and no personal identifying information. After the study has been completed, the record of names and prison ID numbers linked to study ID numbers will be shredded and destroyed.

**Data Sources:** These will include: Structured interviews; HIV Testing Results; and Correctional Databases.

Structured Interviews: All interviews will be conducted during the second and third shifts in private settings. Questions will include information on demographics, history of previous HIV testing experiences, court dates, perceived likelihood of remaining incarcerated, willingness to HIV test now if it were made available, attitudes abut HIV testing in jails and prisons, symptoms of withdrawal, suicidal ideation, psychological distress, and an evaluation of medical ability to consent to testing using the MacArthur Competence Assessment Tool. The forms will be taken to taken to our data management office at the Yale University AIDS Program and entered into a relational database. Record storage conforms to HIPAA regulations. This information will then be used as part of our process measures to examine the frequencies of patients who are medically competent to opt out, their measures of psychological distress and various states of intoxication and withdrawal. This data will be analyzed to examine the time frame by which these outcomes change after the time of incarceration.

HIV Testing Results: Testing results will be recorded by study staff and taken to our data management office at the Yale University AIDS program for entry into an electronic database. Record storage conforms to HIPAA regulations. Research staff will also provide the counselors with the test results. Counselors will arrange for test results to be recorded in Department of Corrections’ medical records. Counselors will refer preliminarily positive subjects for confirmatory testing. All confirmatory positive test results will be reported to the Department of Public Health per state reporting guidelines. Inmates will be informed of these reporting procedures during the consenting process.

Correctional Databases: There is a correctional database that includes all information regarding dates of entry and release from the DOC, medical and psychiatric classification, and pending court cases. We will use this database to access demographic information about subjects for data collection and analysis.

- 1. What methods and procedures will be used to safeguard the confidentiality of subjects and their data?

All research staff members have undertaken HIPAA training and know the importance of maintaining confidentiality of the participants’ information. Study participants will be attended to individually in private space. All data and study instruments, which are recorded by study number only, are maintained in secure password-protected computers and locked cabinets at the Yale AIDS program.

- 1. What mechanisms are in place to ensure proper use and continued protection of these data?

All confidential information (study instruments, test results, etc.) will be recorded with study participant number only and maintained in locked cabinets within our offices at the Yale University AIDS Program and will only be available to be opened by the Study Coordinator, study staff, or the Principal Investigator. Electronic databases will be maintained through password-protected computers and files at the Yale University AIDS Program.

- 1. Describe any limits to confidentiality, e.g., legal requirements for disclosure of reportable diseases, abuse of child or elder, danger to self or others.

Confidentiality has not been a problem in our studies to date; however, we will continue to monitor this as part of our DSMP. Potential instances for breaches of confidentiality include during the recruitment process in jail, interview sessions, delivery of HIV test results, or from data management systems. From our research efforts and provision of clinical care for prisoners in both clinical settings and within the prison system, we have paid careful attention to potential breaches of confidentiality for subjects undergoing interviews. Dr. Altice has written extensively on the legal and ethical issues with regard to conducting clinical trials among prisoners. ^30,31^ All interviews will be conducted individually during the second shift when rooms are more accessible for consent procedures and privacy can be ensured. Appointments will be staggered to avoid participant encounters in the interview room or in the outside hallway.

Any inmate who describes suicidal ideation will be immediately referred to the prison health staff for professional assessment.

Connecticut reporting requirements require that HIV testing is confirmed prior to reporting. Therefore all inmates who test preliminarily positive will undergo confirmatory testing with either a Western blot or immunofluorescent assay (IFA) for confirmation of test results. Only confirmatory results will be reported to the Connecticut Department of Public Health. The DOC counselors will arrange for confirmatory testing and reporting.

- 1. What will be done with the data when the research is completed?

The data will be stored in the manner noted above until completion of the study. Storage will be without individual identifiers. These data will be analyzed so that the findings can contribute to scientific literature that could be used to develop strategies for routine rapid HIV testing in jail settings.

- 1. Will any external individuals or agencies (such as the study sponsor, FDA, etc.) have access to study data?

The HIC will have access to study data.

- 1. If appropriate, has a Certificate of Confidentiality been obtained? For more information, please see the HIC website: <http://www.med.yale.edu/hic/policy/CofC.pdf>

A Certificate of Confidentiality has not been obtained for this study.

1. **Potential Benefits:** Please identify any benefits that may be reasonably expected to result from the research, either to subjects or to society at large.

The most immediate benefit to study participants will be the opportunity to learn their HIV status, receive counseling, and be linked to treatment within the correctional system and hopefully in the community upon release. This is especially important because as many as 50% of individuals who enter correctional settings do not know their HIV status and many of them do not utilize health screening services outside the correctional system. Therefore, the correctional setting presents a unique opportunity to implement screening, prevention, and treatment programs to address patients with or at risk for HIV/AIDS. From a societal perspective, if information gleaned from this study is beneficial and feasible, this may result in further replication of this project elsewhere and benefit a large number of individuals that pass through jails in the U.S. and worldwide.

## Section IX: Research Alternatives and Economic Considerations

1. **Alternatives:** For studies offering treatment, what treatment alternatives are available outside of the research?

*Please note: Some categories of non-treatment research may also require a section outlining alternatives to participation. For example, a study that provides screening for a particular illness or condition should state whether testing is available outside of the research.*

Inmates may request an HIV test outside of this study from one of the DOC HIV counselors.

1. **Payments for participation (Economic Considerations):** Describe any payments that will be made to subjects (including direct monetary payment, payment in the form of a gift, or reimbursement for costs such as travel, parking, childcare, etc.), and the conditions for receiving this compensation.

No payment will be made to subjects.

**Costs for participation (Economic Considerations):** Clearly describe the subject’s costs associated with participation in the research. If it is possible that the subject’s insurance, health plan benefits, or other third party payers will not cover research procedures or tests, this should be indicated. Clearly describe the parts of the research visits (drugs, tests, procedures, etc.) that will be provided at no cost to the subjects.

*Please note: If payment will be prorated for subjects who do not complete the study, this should be clearly explained. If payment is conditional on completing the study, this should be clearly explained.*

There are no costs to the subjects associated with participation in this research study.

1. **In Case of Injury:** Will medical treatment be available if injury occurs? Where and from whom may treatment be obtained? Are there any limits to the treatment being provided? Who will pay for this treatment? How will it be accessed by subjects? (Please refer to the Compensation and Medical Therapy sections of the *HIC Guidance for Investigators Manual* available on the HIC web site: <http://www.med.yale.edu/hic/forms/forms/guidelines.pdf> )

.Any inmate who describes suicidal ideation, or any other acute medical, psychiatric, or other adverse condition, will be immediately referred to the prison health staff for professional assessment. This treatment will be provided at no cost to the subjects.

## Section X: Formatting

Please ensure that your protocol contains the header and footer formatting as demonstrated on this document and version date. Consent, assent, permission, and information sheets must also contain these headers, footers and version dates.

**References**
